# Harmonious color pairings: Insights from human preference and natural hue statistics

**DOI:** 10.1016/j.isci.2026.116038

**Published:** 2026-06-08

**Authors:** Ortensia Forni, Alexandre Darmon, Michael Benzaquen

**Affiliations:** 1 Econophysics Lab, Institut Louis Bachelier, 28 Pl. de la Bourse, Palais Brongniart, 75002 Paris, France; 2 LadHyX UMR CNRS 7646, École Polytechnique, Institut Polytechnique de Paris, 91128 Palaiseau Cedex, France; 3 Art in Research, 33 rue Censier, 75005 Paris, France; 4 Capital Fund Management, 23 Rue de l’Université, 75007 Paris, France

**Keywords:** Cognitive neuroscience, Neuroscience, Applied sciences

## Abstract

While color harmony has long been studied in art and design, consensus remains elusive, as most models rely on qualitative insights or limited datasets. We present a quantitative, data-driven study of color pairing preferences using controlled hue-based palettes in HSL. Participants evaluated combinations of thirteen hues, enabling us to construct a preference matrix and define a combinability index for each color. Preferences are highly hue dependent, thereby challenging classical color harmony theories. Yet, when averaged over hues, statistically meaningful patterns of aesthetic preference emerge, with certain hue separations perceived as more harmonious. Strikingly, these patterns align with hue distributions in natural landscapes, suggesting a statistical correspondence between human color preferences and the color structure in nature. Finally, principal-component analysis of the score matrix reveals two complementary hue groups underlying the global structure of color-pairing preferences. Together, these findings offer a quantitative framework for studying color harmony and potential perceptual and ecological underpinnings.

## Highlights

• Hues that combine well with others are very common in natural scenes
• People tend to prefer hue pairs that are almost opposite on the color wheel
• Natural scene statistics also show predominantly contrasted hue pairings
• Universal harmony rules are not fully supported as preferences depend on hue

## Graphical abstract

**Figure:**
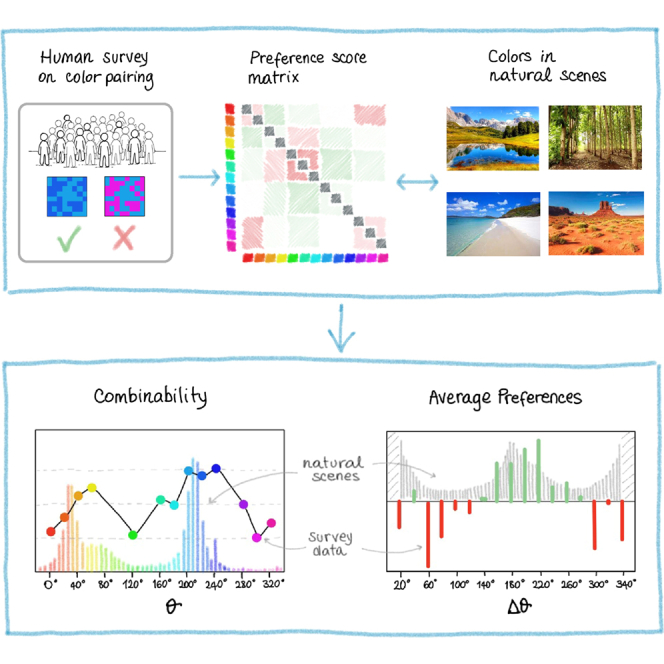

## Introduction

Early efforts to understand the nature of color can be traced back to antiquity with Aristotle’s chromatic theories.^1^ This said, throughout much of history, color was primarily associated with symbolic and religious meanings. In Western civilizations, for example, saturated colors and polychromy were often associated with superficiality, vulgarity, or foreignness, while whiteness was imbued with connotations of purity and reassurance. A scientific turn occurred in the seventeenth century with the emergence of a more intellectual and systematic approach to color, exemplified by the creation of various color charts and tables, such as Richard Waller’s *Catalog of Simple and Mixt Colours*.^2^ A major scientific milestone was reached in 1704 with Newton’s famous treatise, *Opticks*.^3^

Since then, the ambition to build consistent and rigorous color theories has continued to grow, drawing interest from a wide range of disciplines including mathematics, psychology, neuroscience, philosophy, and design, to name a few. A considerable body of research has focused on the construction of well-defined color spaces, whether through strictly mathematical frameworks^4^^,^^5^^,^^6^^,^^7^^,^^8^ or more artistically driven approaches.^9^ One particularly prominent area of color theory that has attracted sustained interest over the centuries is the study of color harmony.^10^^,^^11^^,^^12^^,^^13^ Goethe,^14^ Chevreul,^15^ Ostwald,^16^ among others, first sought to capture the essence of harmonious color pairings by linking aesthetic appreciation to the perceptual distance between colors. More recently, data-driven approaches have gained traction. Some studies have focused on color emotion,^17^^,^^18^^,^^19^^,^^20^^,^^21^ aiming to identify correlations between specific hues and the emotions they trigger. Other works have investigated color preferences in terms of biological adaptations, highlighting marked gender differences,^22^^,^^23^ or through the “Ecological Valence theory”,^24^ which links color preferences to associations with objects perceived positively or negatively. Additional studies have examined how preferences vary with age,^25^^,^^26^ art education,^27^ or seasonality.^28^ Building on these perspectives, researchers have also sought to develop generalizable models of color harmony using machine learning techniques^29^^,^^30^ and to build computational toolkits for extracting image features relevant to aesthetic judgments.^31^ Complementing these scientific developments, a distinctly historical-cultural line of work has emphasized the enduring importance of colors across history and the ways in which the social, moral, and symbolic values attributed to them evolve over time, shaped by historical contexts and collective practices.^32^^,^^33^

In spite of these developments, most approaches to the color pairing problem still lack a truly quantitative foundation. Landmark perception-based studies such as those by Moon and Spencer^34^ or Granger^35^ have produced widely cited harmony principles but were based on a very limited number of survey participants. A more recent and ambitious study was conducted by Nemcsics^36^ in the Coloroid space among Technology and Economics students at Budapest University. However, the high level of complexity in the study—both in the geometrical patterns presented to participants and in the selection of colors combining hue, saturation, and lightness—may limit the interpretability of the results.

In summary, to this day, there remains no clear consensus on the principles underlying color harmony,^37^ thus leaving the topic open to debate and inquiry. Our approach here is inspired by the work of Lakhal et al.,^38^ on the structural complexity of black and white images, in which some of us provided evidence for a certain level of universal quantitative criteria for aesthetic judgment.^39^ Interestingly, the preferred level of complexity correlated strongly with that found in natural images, suggesting that what we find aesthetically pleasing may be shaped by what we are most frequently exposed to. Related work in the color domain has similarly connected aesthetic preferences to statistical regularities in natural images and paintings, pointing to a degree of universality in preferred chromatic compositions.^40^^,^^41^^,^^42^^,^^43^ In line with this approach, we conduct a large-scale survey targeting a sufficiently diverse participant panel and grounded in simple evaluation procedures, aiming both to gain insights into preferences for color pairings and to compare these preferences with color combinations found in natural images.

The remainder of this paper is structured as follows. We first introduce our survey methodology, which is based on direct comparisons of simple color pairs. Next, we analyze the resulting data and propose a metric that quantifies how harmoniously each color pairs with others. We then explore how this metric aligns both with absolute color preferences reported in independent studies and with hue distributions observed in natural images. By averaging across hues, we identify angular distances between colors that are consistently preferred or rejected, and compare these perceptual trends to signals found in natural landscapes. Finally, we examine how different color groups contribute to the emergence of configurations of appreciation and rejection.

## Results and discussion

### Survey

We conduct a large-scale survey in which participants are asked to select their three most and three least preferred color combinations from sets of predefined color palettes, described in details in the following sections. Our study operates within the HSL (Hue, Saturation, and Lightness) color space,^44^ focusing exclusively on the hue component while keeping saturation and lightness fixed at L = 0.5 and S = 0.8.^45^ This choice also supports our large-scale, quantitative objective: by focusing on hue—which is less affected by between-device variability than saturation and lightness—we were able to conduct the survey online and recruit a large, heterogeneous participant pool. Hues are represented as angular values on the HSL color wheel,^46^ with red as reference color positioned at 0°, following the usual convention. We sample 18 equally spaced hues at 20° intervals, starting from 0°, providing a reasonably fine coverage of the full hue wheel while keeping the set manageable for a preference task; then we remove five of them (corresponding to 80°, 100°, 140°, 260°, and 340°) to keep only clearly distinguishable colors (verified across different display devices). Indeed, the HSL color space lacks perceptual uniformity,^47^ as some colors comprised within large angular ranges (such as greens) cannot be unmistakably separated by the eye. In the following, we refer to the 13 retained colors with indices *i* ∈ {1, *…*, 13}, with 0° red corresponding to *i* = 1. We then generate 13 sets of 12 color pairs by taking each color *i* in turn as the reference color, and pairing it with each of the remaining 12 colors *j*, hereafter referred to as the counterpart colors. The color pairs are presented as checkerboards consisting of 8 × 8 square grids, with the reference and counterpart colors distributed in equal amounts, though randomly in space to avoid recognizable patterns that might bias participants’ responses. An example of set with H = 200° blue as reference color is shown in the supplemental information (Figure S1). The survey is structured as follows: for each of the 13 sets, a question is created in which participants are shown, in a random order, one of the sets. Participants are then asked to select the three most and three least harmonious color combinations. To limit survey duration and maintain engagement, each participant was asked to complete 6 randomly assigned questions (out of 13), rather than the full set. The subset of questions varied across participants, ensuring a comparable number of responses for each of the 13 reference hues. The survey was conducted via the Qualtrics platform.^48^ We collected 346 responses from colleagues at École Polytechnique and CFM (France), OIST (Japan), as well as volunteers across Europe who participated without financial incentives. The sample included both male and female participants aged between 20 and 65, mostly with a high level of education but from diverse academic backgrounds.

### Results

For each reference color *i* and counterpart color *j*, we define $f_{ij}^{\text{B}}$ as the frequency with which the color combination (*i*, *j*) was selected among participants as one of the three most harmonious combinations, normalized to the number of survey responses per question. Analogously, $f_{ij}^{\text{W}}$ is the normalized frequency with which (*i*, *j*) was selected as one of the three least harmonious combinations. A high value of $f_{ij}^{\text{B}}$ suggests that color *i* pairs well with color *j*, though a low value does not necessarily imply a negative judgment; it may simply reflect infrequent selection among the top three. Conversely, a high $f_{ij}^{\text{W}}$ suggests unpleasantness while a low value does not imply positive judgment. We thus define the overall score of each color pair as:

(Equation 1) $${\text{S}}_{ij}=f_{ij}^{\text{B}}-f_{ij}^{\text{W}}.$$

**Figure 1:**
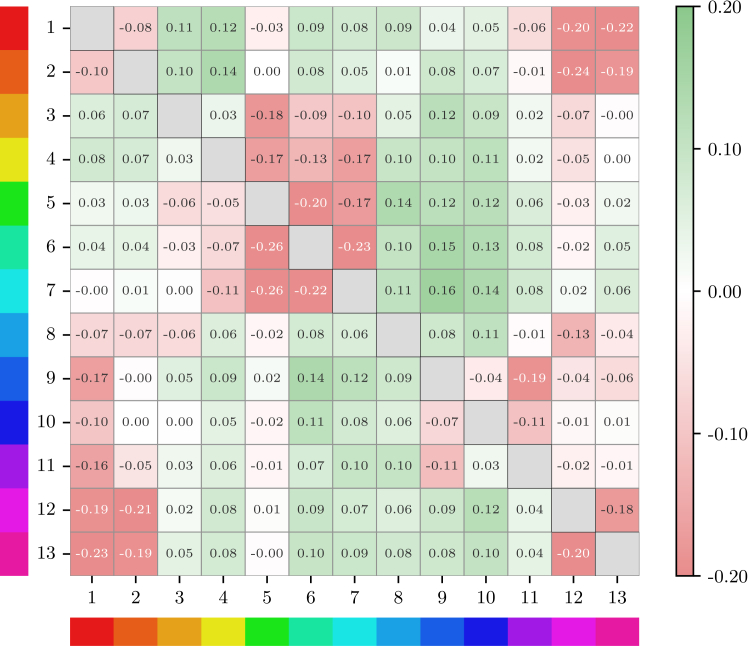
Color pairing survey results Preference matrix S computed from the survey results and Equation 1.

The resulting score matrix S is shown in Figure 1, where the *i*^th^ row corresponds to responses to the question in which color *i* was used as the reference. Note that the rows of the matrix naturally sum to zero (participant answers for each question were only retained if they provided all three best and three worst choices). Remarkably, S reveals an underlying structure, with discernible clusters of color combinations that are perceived as either harmonious or inharmonious. The nature of these clusters is examined in greater depth later in the paper.

Reassuringly, the score matrix appears to be quasi-symmetric (see Figure 4A), indicating that the appreciation or dislike of a color combination does not strongly depend on the specific set in which it is presented to survey participants. In other words, interchanging the reference and counterpart colors leaves the results largely unchanged to first order. That said, the matrix is not perfectly symmetric, and the residual asymmetry carries meaningful information. While the row-wise normalization only allows to capture relative preferences for specific color pairs, the columns reflect judgments of absolute color combinability—that is, the ability of a given color *j* to harmonize with all other colors across the set. For example columns 8, 9, or 10—corresponding to 200°, 220°, and 240° (shades of blue)—are composed of a majority of positive scores. This indicates that these hues tend to be selected more frequently as part of the best than worst combinations. On the contrary, columns of red and purple (columns 1, 12, or 13) carry mostly negative values, suggesting that these colors tend to produce less harmonious combinations when paired with others.

### Combinability index

To quantify how harmoniously a color tends to combine with others, we define, for each color *j*, the combinability index as the sum of its pairing frequencies with all the other colors, across all questions:

(Equation 2) $$\text{C}\left(j\right)=\underset{i\neq j}{\sum }{\text{S}}_{ij}.$$

**Figure 2:**
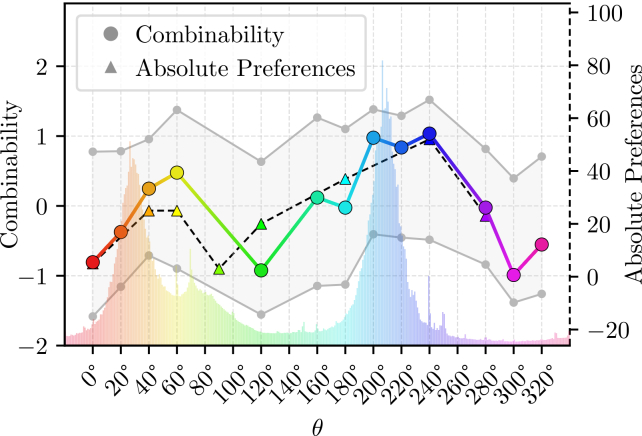
Color combinability Combinability index from Equation 2 (circular markers, solid line), absolute preferences^49^ (triangular markers, dashed line), and hue distribution of 12,000 natural landscape images (background histogram), as a function of hue angular values *θ*. The solid gray lines reflect the contribution of each color to both the best-only and worst-only indices (see main text).

The combinability indices for the 13 colors of our study are plotted in Figure 2. Following the shades of blue (200°, 220°, and 240°) discussed earlier, yellow (60°), closely followed by orange (40°), exhibit the highest combinability indices. Conversely, red (0°), green (120°), and purple (300°) tend to produce combinations that are more frequently perceived as inharmonious. The top and bottom gray lines delimiting the shaded area represent, respectively, the sum C^B^(*j*) and C^W^(*j*) of the best-only frequencies $f_{ij}^{\text{B}}$ and the worst-only frequencies $f_{ij}^{\text{W}}$ for each color *j*. Interestingly, although certain colors like 180° cyan (*j* = 7) have a combinability index close to zero, they show substantial values of C^B^ and C^W^, indicating that such hues are frequently selected by participants—appearing as often in the most appreciated combinations as in the least.

Building on the intuition that combinability might be linked to absolute color preference, we compare our results with findings from the Berkeley Color Project,^49^ which investigated human preferences for eight highly saturated individual colors (triangular markers and dashed line in Figure 2). Remarkably, individual hue preferences closely mirror the combinability index, suggesting that a color’s ability to form harmonious combinations aligns with its overall aesthetic appeal.

Finally, leveraging previous studies that have highlighted a strong correlation between human aesthetic preferences and structures found in nature,^38^^,^^50^^,^^51^ we analyzed a dataset of 12,000 landscape images^52^ spanning various natural biomes, including coasts, deserts, forests, glaciers, and mountains. The count of hue occurrences in the entire database is shown as a background histogram in Figure 2. Strikingly, the peaks and valleys of the distribution match remarkably well that of the combinability index and absolute preferences (maximum for blue and orange-yellow tints, minimum for greens and purples), suggesting that our aesthetic preferences may be influenced by the colors we have most frequently been exposed to. To test the robustness of our findings and avoid potential biases in image selection, we also measured the hue distribution using two additional non-overlapping datasets of 4,319 and 15,501 natural images respectively^53^^,^^54^^,^^55^; the results were found to be virtually identical.

### Preferred combinations

**Figure 3:**
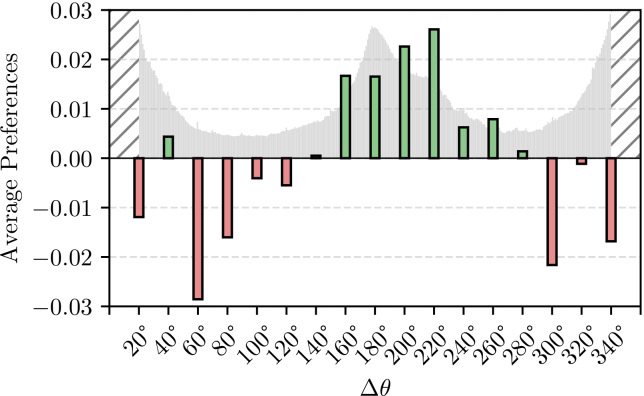
Preferences in angular distance Average preferences for color pairs as function of their angular distance Δ*θ* on the hue wheel (see also Figure S2). The background gray histogram represents the distribution of angular distances between hues found in natural images. The maximum angular distance between hues is Δ*θ* = 180°.

Classical color harmony theories, such as those of Moon and Spencer^34^ or Itten,^11^ tend to rely on a strong assumption of hue independence, meaning that preferences for a given hue pair depend only on the angular distance separating the two colors on the hue wheel, regardless of their absolute positions. To examine the relative positions of the combined colors (*i*, *j*), we rotate the hue wheel in each set such that the reference color is set at 0° (see Figure S3), and assign to each comparison color a value function of its angular distance from the reference. Our findings challenge this hue-independence assumption to a large extent, as discussed in the following sections. Nevertheless, in order to confront our findings with existing theories, we compute, for each angular distance, the average selection frequency across all reference colors. The results are shown in Figure 3, where green bars indicate preference and red bars signify dislike, with 180° corresponding to the maximum angular distance between two hues. Higher preferences are observed in the *contrast* region (between 160° and 220°), flanked by regions of lower preference at smaller angular distances. Such global preference for contrast aligns rather well with the universal harmony model proposed by Moon and Spencer,^34^ which remains influential today.^56^ However, the other preference regions they proposed—coined *similarity* and located near the reference color—are not consistently supported by our hue-averaged data. The universality of such theories is further challenged when examining the standard deviation at each angular distance, shown as error bars in Figure S2 (see supplemental information). The substantial variability observed reflects pronounced differences across individual hue preference wheels (see Figure S3). Consequently, the assumption of hue independence cannot be reasonably upheld. For instance, the preference wheels for green (e), cyan (g), and light purple (l) in Figure S3 display markedly distinct patterns.^57^

To compare our results with hue combination occurrences in natural images, we identify the dominant hue of each landscape in our dataset using a Gaussian kernel density estimation.^58^ For each image, we then count the number of pixels falling at each angular distance from the dominant hue. Summing these counts over the entire dataset yields the gray histogram shown in Figure 3. Note that angular regions between 0°–20° and 340°–360° were excluded from the analysis, as they are artificially overrepresented: since the dominant hue is always aligned to 0°, nearby shades of the same tint are, by construction, more likely to appear. At sufficiently large angular distances from the reference color, dominant hues in natural scenes are most often separated by approximately 180°, suggesting that natural environments are largely characterized by strong color contrasts. The overlay of the empirical preferences and the natural color distance histogram reveals a compelling alignment: the most appreciated color pairs in our survey tend to coincide with those that occur more frequently in nature, while the least appreciated combinations correspond to rarer configurations in natural landscapes. This suggests that our aesthetic preferences for color pairings may be shaped—at least in part—by repeated exposure to common visual patterns in our environment. This finding strongly echoes earlier results by some of us^38^ on black and white image complexity, where aesthetic appeal was likewise found to correlate with structural patterns prevalent in nature. Note that the alignment between preferences and natural color pair occurrences can also be analyzed on a hue-by-hue basis (see Figure S3).

**Figure 4:**
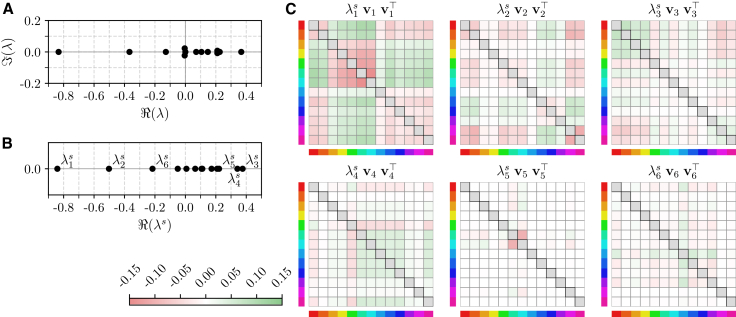
Spectral structure of the preference matrix (A) Eigenvalues of the score matrix S on the complex plane. (B) Eigenvalues of the symmetrized score matrix ${\text{S}}^{s}=\frac{1}{2}\left(\text{S}+{\text{S}}^{⊤}\right)$. (C) Outer products $\lambda_{i}^{\text{s}}{\text{v}}_{i}{{\text{v}}_{i}}^{⊤}$ for the six eigenvalues $\lambda_{i}^{\text{s}}$ with the highest absolute values, where ***v***_***i***_ denotes the eigenvector associated with the *i*^th^ eigenvalue.

To better understand the structure of color pairing preferences—and to assess whether certain colors or groups of colors contribute more significantly to the overall signal—we analyze the symmetric part of the score matrix, defined as ${\text{S}}^{s}=\frac{1}{2}\left(\text{S}+{\text{S}}^{⊤}\right)$, and study its principal components. As previously noted, the original score matrix S exhibits a near-symmetric structure, which is quantitatively supported by the proximity of its eigenvalues to the real axis (see Figure 4A). This observation justifies our focus on the symmetric component *S*^*s*^, the eigenvalues of which are plotted in Figure 4B. In Figure 4C, we display the outer product matrices $\lambda_{i}^{\text{s}}{\text{v}}_{i}{{\text{v}}_{i}}^{⊤}$ corresponding to the six eigenvalues $\lambda_{i}^{\text{s}}$ with the highest absolute values (see Figure 4B), where ***v***_***i***_ denotes the eigenvector associated with the *i*^th^ eigenvalue. The first matrix, associated with the largest eigenvalue, reveals a clear emergence of positive and negative clusters, indicating a division of the hue wheel into two main groups: one spanning from orange to cyan (group 1), and the other from blue to dark orange (group 2). Colors from group 1 tend to combine harmoniously with those from group 2 but not with others within their own group, and vice versa. This structured clustering highlights which segments of the hue spectrum predominantly drive the universal appreciation seen in the contrast region of Figure 3, as well as the adjacent zones of disfavor.

**Figure 5:**
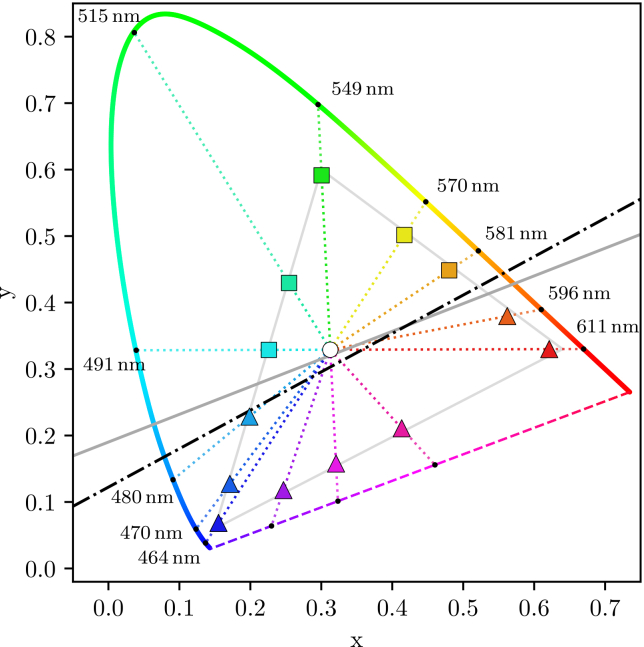
Separation of color groups Representation of our 13 sampled hues in the CIE 1931 xy chromaticity diagram. The outer curved boundary represents the spectral locus and the light-gray triangle corresponds to the sRGB gamut.^60^^,^^61^ Hues belonging to group 1 and group 2 are plotted as squares and triangles respectively, and dotted lines project each color to its dominant wavelength on the spectral locus. The two separating lines are obtained using hard-margin support vector machine (SVM).^62^ The gray one is the standard hard-margin solution, which maximizes the distance to the nearest points of both groups, while the black dash-dotted line is obtained by assigning a weight to the two support vectors on each side that, intuitively, encodes how strongly a color belongs to its group and is defined as $\omega_{i}=\left(\sum_{j:\;S_{ij}^{s}<0}|S_{ij}^{s}|\right)/\left(\sum_{p,q:\;S_{pq}^{s}<0}|S_{pq}^{s}|\right)$.

Interestingly enough, when our color groups are represented in the standard CIE 1931 xy chromaticity diagram^59^ (see Figure 5), they are found to be linearly separable. One can also clearly see that while group 1 includes colors from the central region of the visible spectrum, group 2 is composed of colors from two extremes of such spectrum, together with the purples (which correspond to a mix of blue and red). Finally, note that the decision boundaries shown in Figure 5 pass close to white, at the center of the gamut.

Further structure is visible in the subsequent components. In particular, the second matrix exhibits a more localized negative signal, concentrated among hues from light purple to dark orange (bottom right, red cluster), indicating internal incompatibilities within this range. Additionally, the fifth matrix reveals another pronounced negative contribution, pointing to poor combinability between green and cyan.

### Concluding remarks

Let us summarize the main contributions of this work. We designed and conducted a large-scale survey in which participants were asked to select the most and least appealing color combinations from carefully curated sets. Our first finding was that certain hues—such as blue and yellow—consistently form more harmonious pairings with other colors. We then introduced a combinability index to quantify this behavior, and showed that it correlates well with absolute color preferences reported in an independent study.^49^ More notably, this index also aligns closely with the distribution of hue occurrences in natural landscapes. Shifting focus from individual hues to angular distances between paired colors, we uncovered a robust preference for combinations in the contrast region, centered around complementary hues. This pattern mirrors color distributions commonly found in nature, suggesting that aesthetic appeal may be shaped, at least in part, by frequent exposure to naturally occurring visual stimuli.^38^ That said, our results do not fully align with the universal view of classical color harmony theories,^11^^,^^12^^,^^13^^,^^14^^,^^15^^,^^16^ which assumes that harmony depends mainly on fixed color distances (e.g., simple angular relations) rather than on the absolute colors involved. While contrast is favored on average, preferences vary strongly with the specific hues paired, so fixed-distance rules do not consistently predict human preferences. Finally, a principal component analysis of the score matrix provided further insight, revealing clusters of hues that tend to combine either particularly well or poorly with others, thus offering a more nuanced understanding of the structure underlying human color pairing preferences.

### Limitations of the study

To simplify the interpretation of color combinability and offer an accessible entry point into the question of color harmony, we deliberately limited our analysis to variations in hue only. This choice is also well suited to a large-scale online survey, as hue is comparatively more robust than saturation and lightness to differences across display devices. While this approach offers clarity and tractability, it overlooks the potentially rich interactions with saturation and lightness. A natural direction for future work would be to extend the study to full-color combinations across all HSL dimensions, and to examine how such variations relate to patterns found in natural imagery. Another important avenue lies in broadening the participant panel—not only in size but also in cultural and demographic diversity—to test the generalizability of our findings across different populations. Finally, our use of the HSL color space, though convenient for isolating hue, deserves further discussion. HSL suffers from perceptual non-uniformity and device dependence, limiting its fidelity in capturing human color perception. Future research could explore alternative, perceptually uniform color spaces such as CIELAB (CIE L∗a∗b∗, defined by the *Commission Internationale de l’Éclairage*) and OKLAB.^63^ However, this comes with its own challenges: in such models, hue is no longer a single scalar dimension but arises from interactions between multiple components, complicating direct comparisons.

## Resource availability

### Lead contact

Further information and requests for resources should be directed to and will be fulfilled by the lead contact, Ortensia Forni (ortensia.forni@polytechnique.edu).

### Materials availability

This study did not generate new unique reagents.

### Data and code availability

• Data collected in this study will be shared by the lead contact upon request.
• The dataset of natural scenes used for the comparison with natural hue statistics is publicly available at https://www.kaggle.com/datasets/utkarshsaxenadn/landscape-recognition-image-dataset-12k-images.
• This paper does not report original code.
• Any additional information required to reanalyze the data reported in this article is available from the lead contact upon request.

## Acknowledgments

We are deeply grateful to all the participants of our survey, whose contribution was foundational to this work. We also thank Jean-Philippe Bouchaud, Pierre Bousseyroux, Samy Lakhal, Elia Moretti, and Mirko Polato for fruitful discussions. This research was conducted within the Econophysics & Complex Systems Research Chair, under the aegis of the Fondation du Risque, the 10.13039/501100009454Fondation de l'École Polytechnique, the Ecole polytechnique and Capital Fund Management.

## Author contributions

A.D. and M.B. designed the research; O.F. gathered and analyzed data; O.F., A.D., and M.B. performed research and wrote the paper.

## Declaration of interests

The authors declare no competing interests.

## STAR★Methods

### Key resources table

**Table:** 

REAGENT or RESOURCE	SOURCE	IDENTIFIER
**Deposited data**		

Kaggle landscape recognition image database (12000 images)^52^	Kaggle	https://www.kaggle.com/datasets/utkarshsaxenadn/landscape-recognition-image-dataset-12k-images
Kaggle landscape pictures database^53^	Kaggle	https://www.kaggle.com/datasets/arnaud58/landscape-pictures
Places: An image database for Deep Scene Understanding	Zhou et al.^54^	http://places2.csail.mit.edu/download-private.html

**Software and algorithms**		

Qualtrics xm	Qualtrics^48^	https://www.qualtrics.com/

### Experimental model and study participant details

The survey was conducted online (Qualtrics) and yielded 346 responses from colleagues at École polytechnique and CFM (France), OIST (Japan), and volunteers across Europe; no financial incentives were provided. Participants came from diverse academic backgrounds. Because the study focused on quantifying hue-based pairing preferences rather than demographic effects, we did not collect demographic variables such as age/developmental stage, sex, gender, ancestry, race/ethnicity, or socioeconomic status; therefore, no sex- or gender-based analyses were performed.

### Method details

Our study involved an online behavioral preference task with controlled visual stimuli. We worked in the HSL color space, varying hue only while keeping saturation and lightness fixed (S = 0.8, L = 0.5); hues were sampled every 20° on the HSL wheel and five were removed to retain 13 clearly distinguishable colors across display devices. For each reference hue i, we generated a set of 12 hue pairs (*i*, *j*) and displayed each pair as an 8 × 8 checkerboard with equal amounts of the two colors; square locations were randomized to avoid spatial patterns, and the order of pairs within each question was randomized. Participants selected the three most and three least harmonious combinations per question; to limit duration, each participant completed 6 randomly assigned questions out of 13, with the assignment varying across participants to ensure comparable response counts per reference hue. Selection frequencies were normalized by the number of responses per question to define $f_{ij}^{\text{B}}$, $f_{ij}^{\text{W}}$, and the score ${\text{S}}_{ij}$ (defined by Equation 1). We then analyzed the score matrix (including its symmetric part) via eigen-decomposition/principal component analysis to identify structure and hue groups. To compare preferences with natural statistics, we computed hue distributions from a 12,000-image landscape dataset and two additional non-overlapping natural-image datasets (4,319 and 15,501 images), using kernel density estimation to identify dominant hues and angular-distance histograms; these datasets and methods are cited in the references (e.g., Kaggle datasets,^52^^,^^53^ Places database,^54^ KDE^58^).

### Quantification and statistical analysis

Quantification is based on choice frequencies in an online preference task. For each reference hue *i*, participants evaluated 12 pairings (*i*, *j*) and selected the three most and three least harmonious; we compute normalized frequencies $f_{ij}^{\text{B}}$, $f_{ij}^{\text{W}}$(normalized by the number of valid responses to that question) and the score *S*_*ij*_ defined by Equation 1, from which we derive a combinability index per hue. Note that the number of valid responses contributing to a given question/pairing varies across questions because each participant answered a randomly assigned subset of 6 questions out of 13; total completed surveys =346. The score matrix is analyzed by eigen-decomposition/principal component analysis (PCA), and natural-image hue statistics are estimated via kernel density estimation and angular-separation histograms (see STAR Methods). Question assignment and stimulus order were randomized. No *a priori* sample-size estimation was performed. Analyses were performed in Python and the survey was administered via Qualtrics.
