# Supplementary material for: Harmonious color pairings: Insights from human preference and natural hue statistics
Source: iScience. 2026 Jun 8;29(6):116038. doi: 10.1016/j.isci.2026.116038 (PMC13264017; doi:10.1016/j.isci.2026.116038)
Supplement: Document S1. Figures S1–S3 [file mmc1.pdf]

iScience, Volume *29*

Supplemental information

Harmonious color pairings: Insights from human preference and natural hue statistics

Ortensia Forni, Alexandre Darmon, and Michael Benzaquen

SUPPLEMENTARY INFORMATION

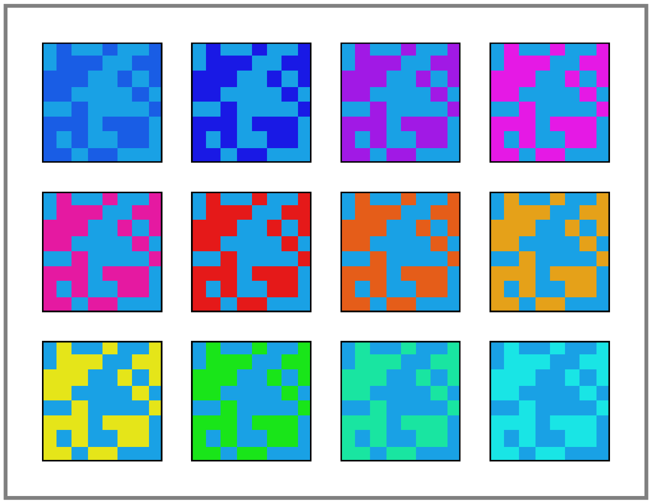

FIG. S1. Example of a survey set with H = 200° as reference color, related to Fig. 1.

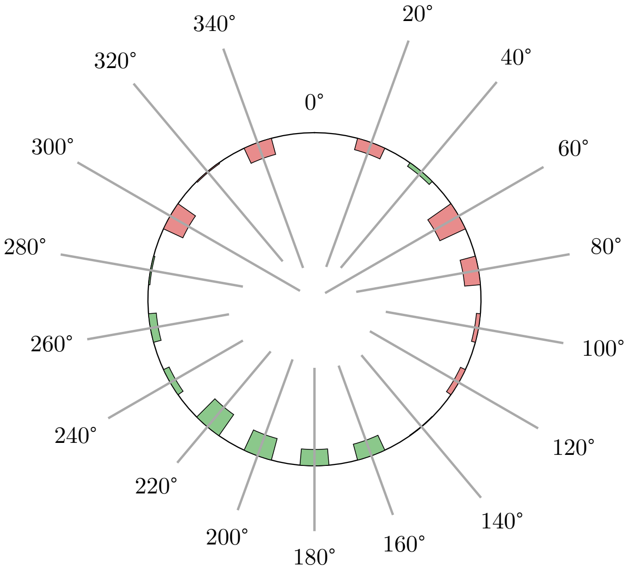

FIG. S2. Average preferences for color pairs as function of their angular distance–as in Fig. 3–presented on a wheel with error bars signifying standard deviations. Green corresponds to positive values, while red indicates negative ones.

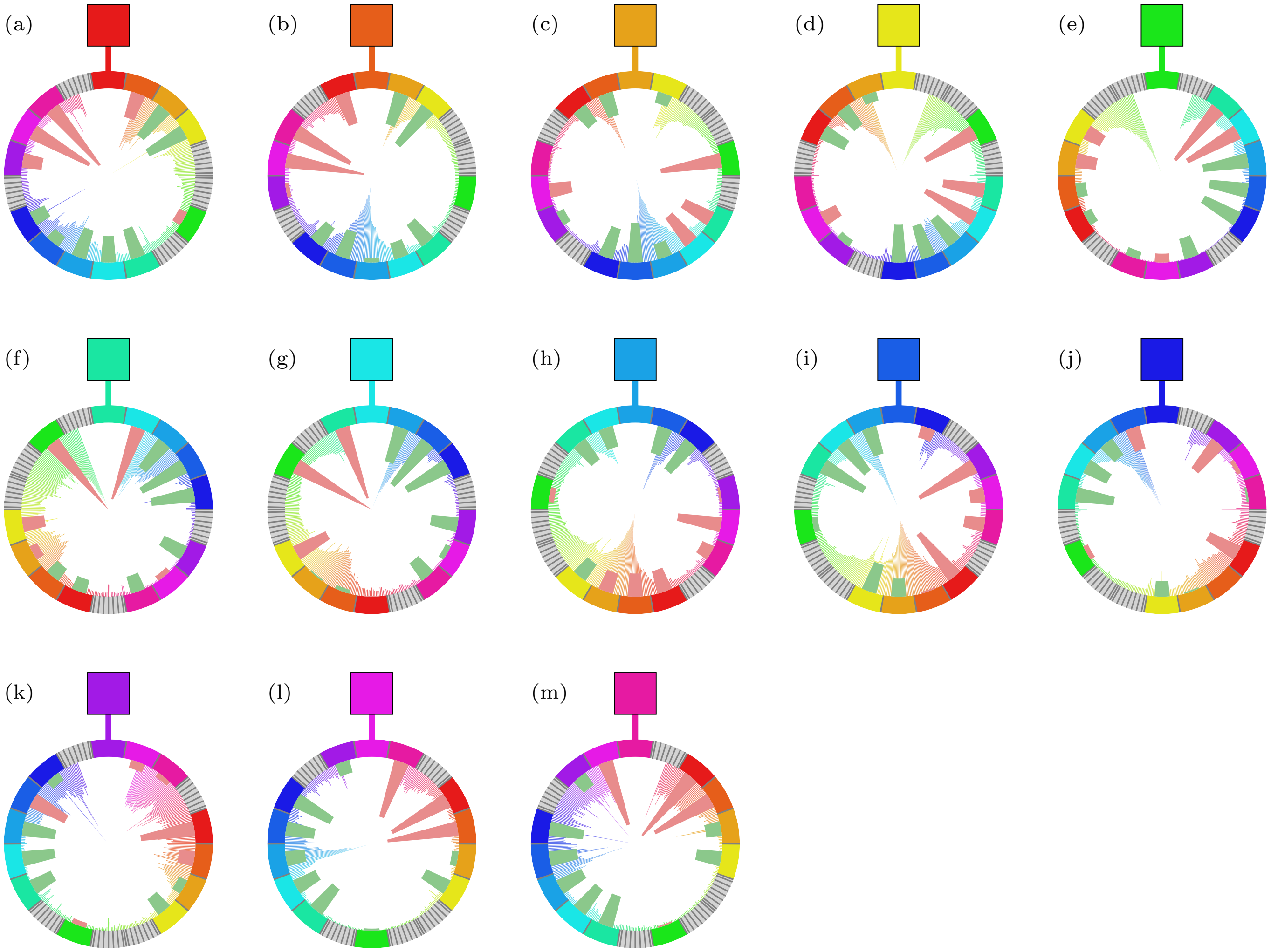

FIG. S3. Color pair preferences for each of the 13 hues considered, related to Fig. 3. Green corresponds to positive values, while red indicates negative ones. The background histograms show the distribution of angular distances relative to the reference hue, calculated over subsets of natural images where this hue appears as the dominant color.
